# Atrial volume reduction correlates with early improvement in hemorrhage-associated normal pressure hydrocephalus—a 3D computed tomography volumetric study

**DOI:** 10.3389/fneur.2026.1753739

**Published:** 2026-07-01

**Authors:** Yanming Huang, Hengjie Mo, Tianqing Liu, Zhiqin Lin, Celin Guan, Shuanglin Que, Yuanxiang Lin

**Affiliations:** 1 Department of Neurosurgery, Neurosurgery Research Institute, The First Affiliated Hospital of Fujian Medical University, Fuzhou, Fujian, China; 2 Department of Neurosurgery, Longyan First Affiliated Hospital of Fujian Medical University, Longyan, Fujian, China; 3 Department of Neurosurgery, National Regional Medical Center, Binhai Campus of the First Affiliated Hospital, Fujian Medical University, Fuzhou, Fujian, China; 4 Fujian Provincial Clinical Research Center for Neurological Diseases, First Affiliated Hospital, Fujian Medical University, Fuzhou, Fujian, China; 5 Clinical Research and Translation Center, The First Affiliated Hospital, Fujian Medical University, Fuzhou, Fujian, China

**Keywords:** hemorrhage-associated normal pressure hydrocephalus, lateral ventricles, computed tomography, 3D Slicer, treatment outcome, ventriculoperitoneal shunt

## Abstract

**Background:**

Hemorrhage-associated normal pressure hydrocephalus (HANPH) is a secondary hydrocephalus with limited research on ventricular volume changes and their correlation with clinical outcomes after ventriculoperitoneal shunting (VPS).

**Methods and materials:**

This retrospective pre-post within-subject study included 180 adult HANPH patients who underwent VPS. CT scans were obtained preoperatively and 2 weeks post-VPS. Lateral ventricular subregions (frontal horn, body, atrium, occipital horn, temporal horn) were segmented and measured using 3D Slicer. Gait ability, modified Rankin Scale (mRS), and Barthel Index (BI) were assessed. Early clinical improvement was defined as >25% gait improvement, >1-point mRS reduction, or >10-point BI increase.

**Results:**

Significant reductions in absolute volumes of lateral ventricular subregions were observed post-VPS. Postoperatively, the relative volume of the lateral body of the lateral ventricle increased, while that of other subregions decreased. Reductions in the absolute volumes of the frontal horn, temporal horn, and atrium negatively correlated with early clinical improvement. Logistic regression revealed that for every 5‰ reduction in volume of these subregions, the likelihood of clinical improvement significantly increased (180.3, 340.9, and 504.2%, respectively).

**Conclusion:**

The reduction in atrial volume of the lateral ventricle is crucial for early clinical improvement in HANPH post-VPS. Catheter placement in the occipital horn may optimize outcomes by effectively reducing atrial volume and minimizing risks. Prospective studies are warranted for validation.

## 1 Introduction

Hemorrhage-associated normal pressure hydrocephalus (HANPH) refers to hydrocephalus caused by conditions related to brain hemorrhage, such as subarachnoid hemorrhage, arteriovenous malformations, and hypertensive intracerebral hemorrhage (1, 2), whose typical clinical presentation includes gait disturbance, cognitive impairment, and urinary incontinence. Imaging of HANPH often reveals ventricular enlargement, while lumbar puncture shows normal cerebrospinal fluid pressure, consistent with the diagnostic criteria for normal pressure hydrocephalus.

Currently, the use of ventricular volume in hydrocephalus research is becoming popular, with most studies focusing on idiopathic normal pressure hydrocephalus (iNPH) (3–5). However, research on secondary hydrocephalus, particularly HANPH, remains limited. As a result, there is a lack of systematic data supporting the relationship between postoperative clinical improvement and changes in ventricular volume in HANPH. Most studies currently rely on follow-up results from idiopathic hydrocephalus, but these findings are inconsistent (3–5). Additionally, existing research often fails to analyze the left and right ventricles and their five subregions in detail, which may obscure important localized pathophysiological changes.

Accurately measuring the volumes of subregions within the lateral ventricles using Computed Tomography (CT) data has become a critical technical challenge in this field (6). Exploring the patterns of ventricular volume changes in HANPH and their relationship with early clinical symptom improvement has, therefore, become a key focus of neurosurgical research.

By combining these volumetric data with functional status and gait assessments, the study aims to determine which specific ventricular subregion volume changes are most critically associated with early clinical improvement. We hypothesize that the reduction in volume of specific lateral ventricular subregions (particularly the atrium) following VPS is correlated with early clinical improvement in HANPH patients. The goal is to provide new insights and a theoretical foundation for clinical intervention and treatment strategies for HANPH.

## 2 Materials and methods

### 2.1 Clinical study population

This study was a retrospective, self-controlled investigation. The participants were adult patients diagnosed with HANPH who underwent VPS at the Department of Neurosurgery, Longyan First Affiliated Hospital of Fujian Medical University, between September 2015 and March 2023.

#### 2.1.1 Ethical approval

The study was approved by the institutional ethics committee (Approval Number: LYREC2022-025-01). The requirement for individual informed consent was waived due to the retrospective nature of the study.

#### 2.1.2 Study groups and rationale for grouping

Patients were categorized into four groups based on two key clinical factors that were hypothesized to potentially influence ventricular geometry and the response to VPS:

Whether they had undergone a preoperative decompressive craniectomy (DC) for the initial brain hemorrhage. This factor significantly alters intracranial biomechanics.

The side of ventricular catheter placement during the VPS surgery (Right-Side Catheterization, RSC; or Left-Side Catheterization, LSC). This factor allows for the investigation of ipsilateral vs. contralateral ventricular volume changes.

The resulting groups were:

Group 1: HANPH, non-DC, right-side catheterization (RSC).

Group 2: HANPH, non-DC, left-side catheterization (LSC).

Group 3: HANPH, DC, right-side catheterization (RSC).

Group 4: HANPH, DC, left-side catheterization (LSC).

This grouping strategy was employed to enable a detailed analysis of how these specific clinical scenarios affect volumetric changes and clinical outcomes. It is important to note that this was a retrospective study; therefore, the group assignment was not randomized but was determined by the patients’ clinical course and surgical decisions. The baseline demographic characteristics of these groups are detailed in the Results section.

### 2.2 Inclusion criteria

Adults (≥18 years) with a history of brain hemorrhage.

Presence of at least one clinical symptom of normal pressure hydrocephalus (e.g., gait disturbance, cognitive impairment, urinary incontinence).

Pre-and postoperative CT scans showing progressive ventricular enlargement without obstruction (6).

Lumbar puncture confirming normal cerebrospinal fluid pressure (70–180 mmH₂O), without the influence of intracranial pressure-lowering medication.

Ventriculoperitoneal shunt (VPS) valve pressure set 20 mmH₂O below the lumbar puncture pressure.

Correct ventricular catheter placement at the frontal horn of the lateral ventricle.

Prior decompressive craniectomy, if applicable, addressed before or during VPS surgery.

### 2.3 Exclusion criteria

Incomplete or substandard imaging data.

Hydrocephalus from non-hemorrhagic causes (e.g., trauma, infection, tumor).

Severe comorbid systemic or neurological diseases that could confound functional assessment.

Major perioperative complications (e.g., infection, shunt failure requiring revision).

Radiographic or clinical evidence of extreme brain compliance (e.g., slit ventricle syndrome).

## 3 Data collection

### 3.1 CT data collection

CT scans were performed using a GE Discovery750 CT scanner (General Electric Company, Fairfield, CT, United States) with the following parameters: slice thickness of 1.25 mm, inter-slice spacing of 1.25 mm, and scan coverage extending from the top of the skull to the mandibular angle. The images were saved in Digital Imaging and Communications in Medicine (DICOM) format.

The DICOM data were categorized into four groups as previously described:

- HANPH, non-DC, right-side catheterization (RSC)
- HANPH, non-DC, left-side catheterization (LSC)
- HANPH, DC, right-side catheterization (RSC)
- HANPH, DC, left-side catheterization (LSC)

CT DICOM data were collected at two time points: within 1 week before and 2 weeks after VPS surgery.

### 3.2 Clinical data collection

Demographic data (gender, age) and relevant medical history were collected. Clinical assessments, including gait ability, functional status measured by the modified Rankin Scale (mRS) (7), and self-care capability assessed by the Barthel Index (BI) (8), were recorded for the week prior to and 2 weeks following VPS surgery.

Gait ability was objectively assessed by counting the number of steps and the time required to walk 10 meters in a straight line on a flat surface (9). Patients were instructed to walk as quickly as safely possible. If a patient was unable to walk, the values were recorded as 100 steps and 600 s, respectively, for analytical purposes (3).

Early clinical improvement was defined as a binary outcome (yes/no) based on the following criteria observed at 2 weeks post-VPS compared to the preoperative baseline, meeting one of these criteria is Early clinical improvement: (1) A greater than 25% improvement in gait ability (either in time or steps), OR (2) A reduction of more than 1 point on the mRS, OR (3) An increase of more than 10 points on the BI. To identify which specific functional domain drove the observed associations with ventricular volume changes, we additionally performed separate logistic regression analyses for each individual outcome component: (i) gait improvement alone (>25% improvement in time or steps), (ii) mRS improvement alone (>1-point reduction), and (iii) BI improvement alone (>10-point increase). These analyses were adjusted for the same clinical covariates as the primary multivariable model (age, hemorrhage type, baseline mRS, and DC status).

### 3.3 Measurement of cranial and lateral ventricular subregion volumes using 3D slicer

Volumetric segmentation was performed using 3D Slicer (version 4.11^1^). Intracranial volume (IV) was measured using the Swiss Skull Stripper module with automated extraction, followed by manual quality control to correct misclassifications.

**Figure 1:**
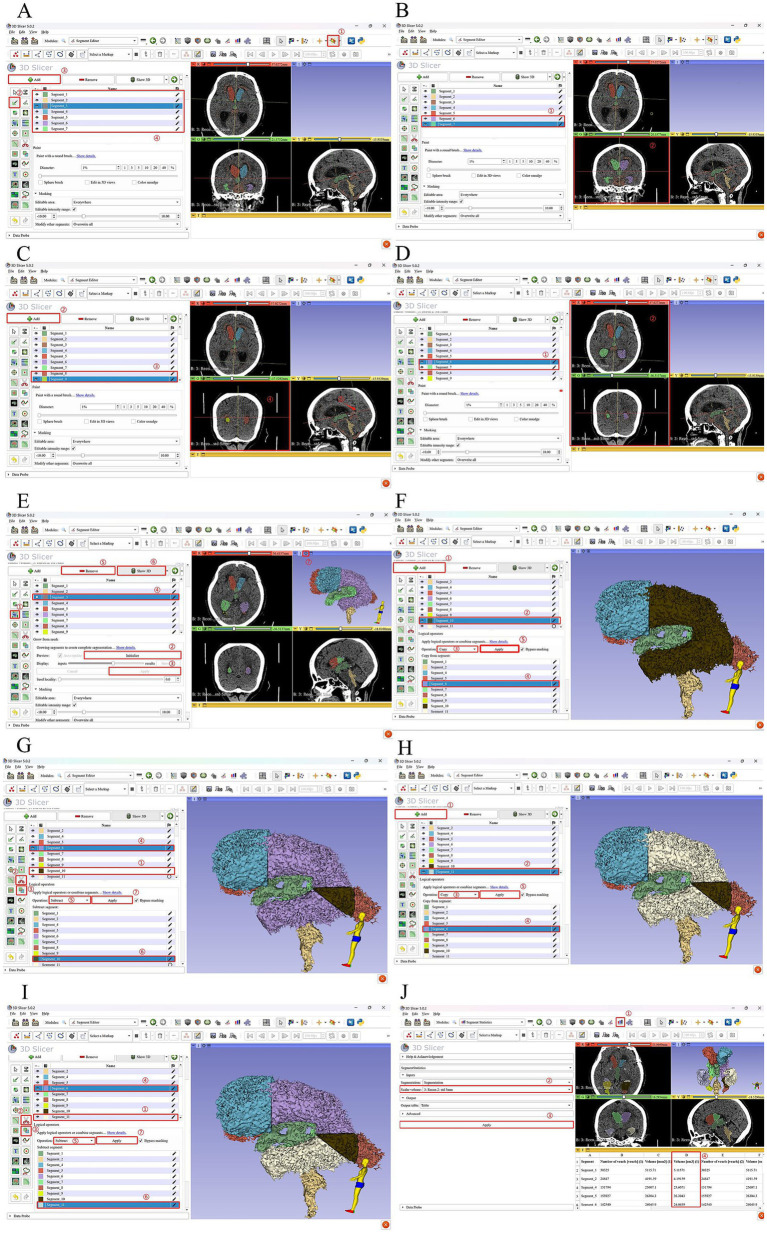
Schematic diagram of lateral ventricle subregions volume measured by Segment Editor. Mask frontal horn and temporal horn of lateral ventricle **(A)**; mask body of lateral ventricle **(B)**; mask occipital horn of lateral ventricle **(C)**; mask atrium of lateral ventricle **(D)**; expand segments by Grow from Seeds **(E)**; separate atrium of left lateral ventricle **(F,G)**; separate temporal horn and body of left lateral ventricle **(H,I)**; measurement of lateral ventricle subregions volume by Segment Statistics **(J)**.

Lateral ventricular subregion segmentation followed a semi-automated protocol combining manual initialization with automated expansion. Specifically: (1) Manual initialization: Two neurosurgeons independently placed seed points within each lateral ventricle subregion (frontal horn, body, atrium, occipital horn, temporal horn) on representative axial, sagittal, and coronal slices using the Segment Editor module; (2) Automated expansion: The “Grow from Seeds” tool automatically expanded segmentations based on CSF intensity thresholding (0–20 HU); (3) Manual refinement: Paint and erase tools were used to correct boundaries and ensure anatomical accuracy. The atrium was delineated using its bend point as the apex and the sagittal projection line against the occipital horn as the boundary (Figure 1G). Left and right ventricles were segmented separately. All segmentations were reviewed in 3D rendering view. The “Segment Statistics” module computed absolute volumes (mm^3^) (10). Relative volumes were calculated as (subregional volume / intracranial volume) × 100%. The detail information can be seen in the Supplementary methods (Figures 1A–G).

### 3.4 Statistical analysis

The intracranial volume (IV) was measured independently by a neurosurgeon, and the data were used for statistical analysis. The volumes of the subregions of the lateral ventricle were independently measured in a double-blind manner by two neurosurgeons proficient in using 3D Slicer. The average of their measurements was used for subsequent analysis. The detail information can be seen in the Supplementary methods. Reliability of volumetric measurements was quantified using intraclass correlation coefficients (ICC). Inter-rater reliability was assessed by having two independent neurosurgeons measure lateral ventricular subregion volumes in 30 randomly selected cases (approximately 17% of the total cohort). Intra-rater reliability was evaluated by having one rater repeat measurements after a two-week washout period. ICC values were interpreted as follows: ≥0.90 excellent, 0.75–0.89 good, 0.50–0.74 moderate, and <0.50 poor.

## 4 Results

### 4.1 Baseline characteristics of the study population

A total of 180 HANPH patients were included in the study and stratified into the four pre-defined groups. The baseline demographic characteristics are comprehensively summarized in Table 1. The groups showed differences in baseline characteristics, which reflect the non-randomized, retrospective nature of the study and the underlying clinical reasons for group assignment (e.g., the side of cranial defect or primary hemorrhage influencing the side of catheter placement). A post-hoc power analysis confirmed that the final sample size (*N* = 180) provided sufficient statistical power (>90%) to detect the clinically relevant associations reported in this study. Reliability analyses demonstrated excellent agreement for lateral ventricular subregion volume measurements. The inter-rater ICC was 0.92 (95% CI: 0.87–0.96), and the intra-rater ICC was 0.95 (95% CI: 0.91–0.98), confirming high reproducibility of the 3D Slicer-based segmentation protocol.

**Table 1:** Baseline characteristics of the study population.

Group	HANPH (non-DC, RSC) (*n* = 72)	HANPH (non-DC, LSC) (*n* = 39)	HANPH (DC, LSC) (*n* = 33)	HANPH, (DC, RSC) (*n* = 36)
Age (years), Mean ± SD	63.00 ± 9.49	56.05 ± 17.76	47.00 ± 9.53	51.05 ± 9.56
Sex Female, *n* (%)	42 (58.33%)	21 (53.85%)	12 (36.36%)	14 (38.9%)

### 4.2 3D modeling diagram of the subregions of the lateral ventricle in HANPH

**Figure 2:**
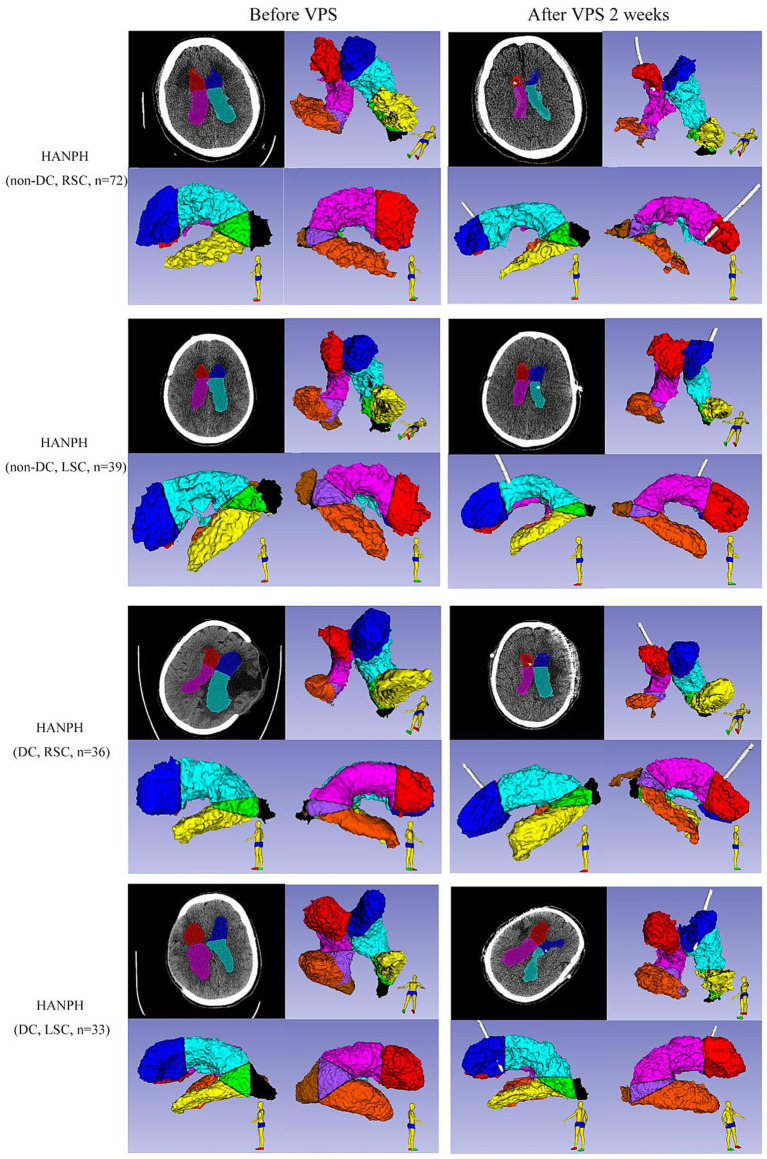
3D Slicer reconstructed lateral ventricle subregions models for HANPH. Lateral ventricle subregions before and after VPS in HANPH were reconstructed by 3D Slicer. RFV was red, RBV was pink, RAV was purple, ROV was brown, RTV was orange, LFV was dark blue, LBV was light blue, LAV was green, LOV was black, LTV was yellow, and VPS tube was white.

The subregions of the lateral ventricle are divided into the frontal horn, body, atrium, occipital horn, and temporal horn, with separate sections for the left and right ventricles, totaling ten subregions. For example, the right frontal horn of the lateral ventricle (RFV), and similarly for other subregions (abbreviations for the subregions of the left and right ventricles are provided in Supplementary Table S1). The 3D modeling diagrams of the subregions for each group are shown in Figure 2, which visually demonstrates the segmentation outcomes and the relationship with the VPS catheter.

### 4.3 Comparison of absolute and relative volumes of lateral ventricular subregions in HANPH before and after VPS

**Figure 3:**
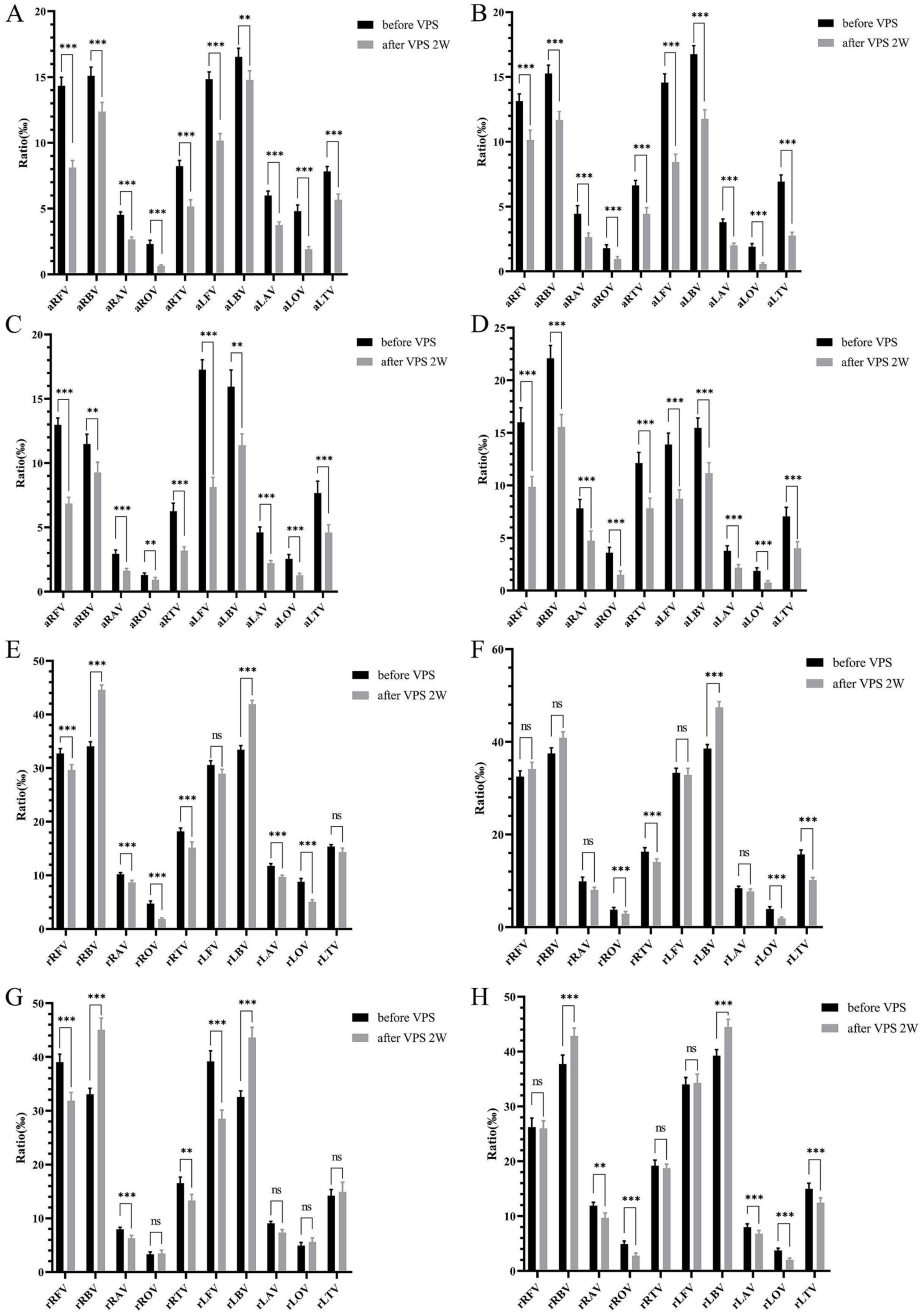
Comparison of ventricular volume in the lateral ventricle subregions before and after VPS in HANPH. It shows differences in the absolute ventricular volume of the lateral ventricle subregions before and after VPS in HANPH (non-DC, RSC) **(A)**, HANPH (non-DC, LSC) **(B)**, HANPH (DC, RSC) **(C)**, and HANPH (DC, LSC) **(D)**. Additionally, differences in the relative ventricular volume of these subregions before and after VPS are observed in HANPH (non-DC, RSC) **(E)**, HANPH (non-DC, LSC) **(F)**, HANPH (DC, RSC) **(G)**, and HANPH (DC, LSC) **(H)**. ns: *p* ≥ 0.05, *p < 0.05, **p < 0.01 and ***p < 0.001.

Following VPS surgery, all patient groups exhibited a significant reduction in the absolute volumes of all lateral ventricular subregions (Figures 3A–D). In most groups, the subregional volume distribution consistently followed the pattern: body > frontal horn > temporal horn > atrium > occipital horn, both before and after surgery.

Notably, in the HANPH (non-DC) group, preoperative subregional volumes were relatively symmetrical between hemispheres. After surgery, the catheterized side demonstrated smaller absolute volumes than the contralateral side (Figures 3A,B). In the HANPH (DC) group, preoperative volumes were larger on the decompressive craniectomy (DC) side. Postoperatively, this relationship reversed, with the catheterized side showing smaller volumes than the DC side (Figures 3C,D).

Regarding relative volumes, the lateral body region demonstrated a significant postoperative increase across all groups, with the rLBV increase being statistically significant (Figures 3E–H). Other subregions showed either decreases or non-significant changes. The relative volume distribution maintained the same pattern as absolute volumes in most groups. No significant interhemispheric differences in relative volume changes were observed during the perioperative period in either the HANPH (non-DC) or HANPH (DC) groups (Figures 3E–H).

Complete quantitative data, including means, 95% confidence intervals, medians, ranges, and statistical test results, are available in Supplementary Table S2.

### 4.4 Relationship between differences in lateral ventricular subregion volumes before and after VPS and early clinical improvement in HANPH

**Figure 4:**
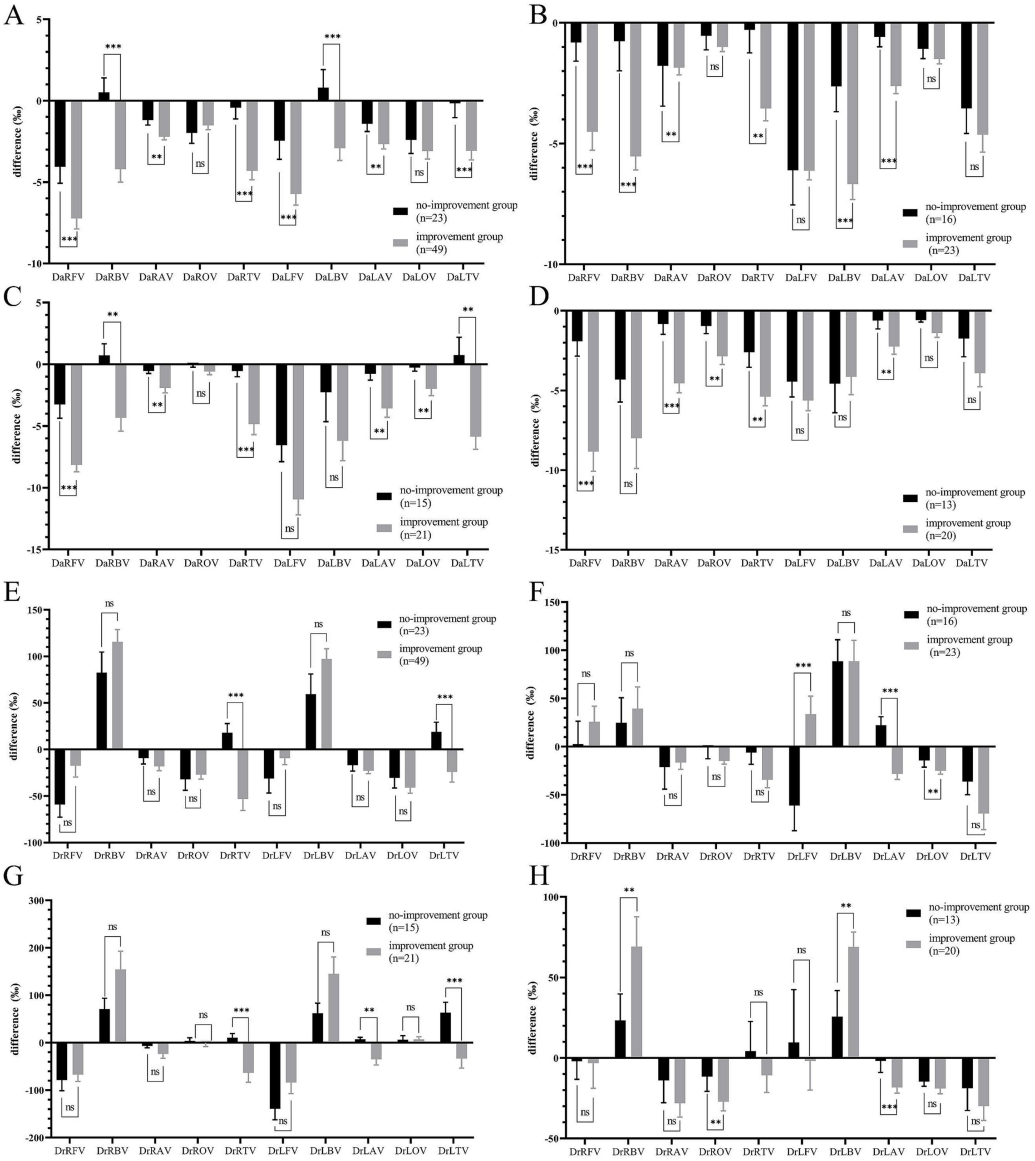
The relationship between the early clinical symptom improvement and differences in ventricular volume of the lateral ventricle subregions before and after VPS in HANPH. It shows that the early clinical improvement is associated with differences in the absolute ventricular volume of the lateral ventricle subregions in HANPH (non-DC, RSC) **(A)**, HANPH (non-DC, LSC) **(B)**, HANPH (DC, RSC) **(C)**, and HANPH (DC, LSC) **(D)**. Additionally, there is a relationship with differences in the relative ventricular volume of subregions in HANPH (non-DC, RSC) **(E)**, HANPH (non-DC, LSC) **(F)**, HANPH (DC, RSC) **(G)**, and HANPH (DC, LSC) **(H)**. ns: *p* ≥ 0.05, *p < 0.05, **p < 0.01 and ***p < 0.001.

In all four groups, the absolute volume differences of the lateral ventricular subregions in the improvement group were negative, and most of the differences in the non-improvement group were also negative, indicating that, the absolute volumes of the lateral ventricular subregions postoperatively were smaller than those before VPS. Additionally, the reduction in the differences in the improvement group was greater than in the non-improvement group. Statistical differences were observed for DaRFV, DaRAV, DaRTV, and DaLAV across all groups (Figures 4A–D). In the improvement group, the magnitude of the absolute volume differences decreased in the following order: frontal horn, body, temporal horn, atrium, and occipital horn, while no clear pattern was observed in the non-improvement group. For improvement group, the reduction in the absolute volume difference on the catheter side was greater than the reduction on the opposite side in the HANPH (non-DC) group (Figures 4A,B), while the reduction in the difference on the catheter side was smaller than the reduction on the opposite side in the HANPH (DC) group (Figures 4C,D). On the other hand, for the non-improvement group, no distinct pattern or trend was observed.

No statistically significant differences were observed in the relative volume differences of the lateral ventricular subregions between the improvement and non-improvement groups across all four groups. However, the reduction in the body of the lateral ventricle was generally smaller in the improvement group compared to the non-improvement group (Figures 4E–H).

Given the statistical differences in DaRFV, DaRAV, DaRTV, and DaLAV across all four groups, a binary logistic regression analysis was performed to assess the relationship between these four indicators and early clinical improvement (Table 2). The results showed that for every 5‰ decrease in DaRFV, the likelihood of early clinical improvement in HANPH patients increased by 180.3% (OR = 2.803, 95% CI: 1.881–4.177); for every 5‰ decrease in DaRAV, the likelihood increased by 121.1% (OR = 2.211, 95% CI: 1.165–4.195); for every 5‰ decrease in DaRTV, the likelihood increased by 340.9% (OR = 4.409, 95% CI: 2.622–7.416); and for every 5‰ decrease in DaLAV, the likelihood increased by 504.2% (OR = 6.042, 95% CI: 2.889–12.637). These large OR values reflect the small unit of measurement (5‰ of total intracranial volume); when converted to a clinically more interpretable scale of 1% (10‰) reduction, the corresponding ORs were 1.52 for DaRFV, 1.32 for DaRAV, 2.10 for DaRTV, and 2.46 for DaLAV. The wide confidence intervals, particularly for DaLAV, reflect the limited number of patients in the higher reduction categories (e.g., only 3 patients had ≥20‰ reduction in DaRFV), underscoring the need for cautious interpretation and larger validation cohorts.

**Table 2:** Binary logistic regression of difference in absolute ventricular volume of the lateral ventricle subregions before and after VPS in HANPH and early clinical symptom improvement.

Difference in absolute ventricular volume of the lateral ventricle subregions before and after VPS	Non-improvement (*n* = 67)	Improvement (*n* = 113)	*B*	Standard error	Wald	OR	95% CI	
DaRFV			1.031	0.204	25.630	2.803***	1.881	4.177
Increased	20	4						
Decreased < 5‰	23	29						
Decreased < 10‰	22	52						
Decreased < 15‰	1	20						
Decreased < 20‰	0	6						
Decreased ≥ 20‰	1	2						
DaRAV			0.793	0.327	5.896	2.211*	1.165	4.195
Increased	20	7						
Decreased < 5‰	44	94						
Decreased < 10‰	0	12						
Decreased < 15‰	1	0						
Decreased < 20‰	2	0						
DaRTV			1.484	0.265	31.290	4.409***	2.622	7.416
Increased	32	10						
Decreased < 5‰	27	54						
Decreased < 10‰	8	43						
Decreased < 15‰	0	6						
DaLAV			1.799	0.377	22.824	6.042***	2.889	12.637
Increased	25	8						
Decreased < 5‰	40	86						
Decreased < 10‰	2	19						

*p < 0.05, ***p < 0.001.

After adjustment for age, hemorrhage type, baseline mRS, and DC status, the multivariable model yielded attenuated but still statistically significant associations: for every 5‰ decrease in DaRFV, OR = 2.341 (95% CI: 1.523–3.599, *p* < 0.001); for DaRTV, OR = 3.652 (95% CI: 2.134–6.251, *p* < 0.001); and for DaLAV, OR = 4.891 (95% CI: 2.312–10.348, *p* < 0.001). The association for DaRAV became marginally non-significant after adjustment (OR = 1.876, 95% CI: 0.987–3.564, *p* = 0.055). These findings indicate that volume reductions in the frontal horn, temporal horn, and atrium remain independently predictive of early clinical improvement after controlling for key clinical confounders (Table 3).

**Table 3:** Difference in absolute ventricular volume after adjusted for age, hemorrhage type, baseline mRS, and DC status.

Difference in absolute ventricular volume	Multivariable OR (95% CI)*	*p* value
DaRFV (per 5‰ decrease)	2.341 (1.523–3.599)	<0.001
DaRAV (per 5‰ decrease)	1.876 (0.987–3.564)	0.055
DaRTV (per 5‰ decrease)	3.652 (2.134–6.251)	<0.001
DaLAV (per 5‰ decrease)	4.891 (2.312–10.348)	<0.001

*Adjusted for age, hemorrhage type, baseline mRS, and DC status.

### 4.5 Sensitivity analysis of atrium boundary definition

The correlation between the primary and alternative atrium volume measurements was strong (Pearson *r* = 0.89, 95% CI: 0.81–0.94, *p* < 0.001). Logistic regression using the alternative atrium volume differences yielded consistent results: for every 5‰ decrease in the alternative DaRAV, the likelihood of early clinical improvement increased by 108.9% (OR = 2.089, 95% CI: 1.098–3.973, *p* = 0.024); for every 5‰ decrease in the alternative DaLAV, the likelihood increased by 471.2% (OR = 5.712, 95% CI: 2.734–11.934, *p* < 0.001). These findings confirm that the association between atrial volume reduction and early clinical improvement is robust across different anatomical boundary definitions.

## 5 Discussion

**Figure 5:**
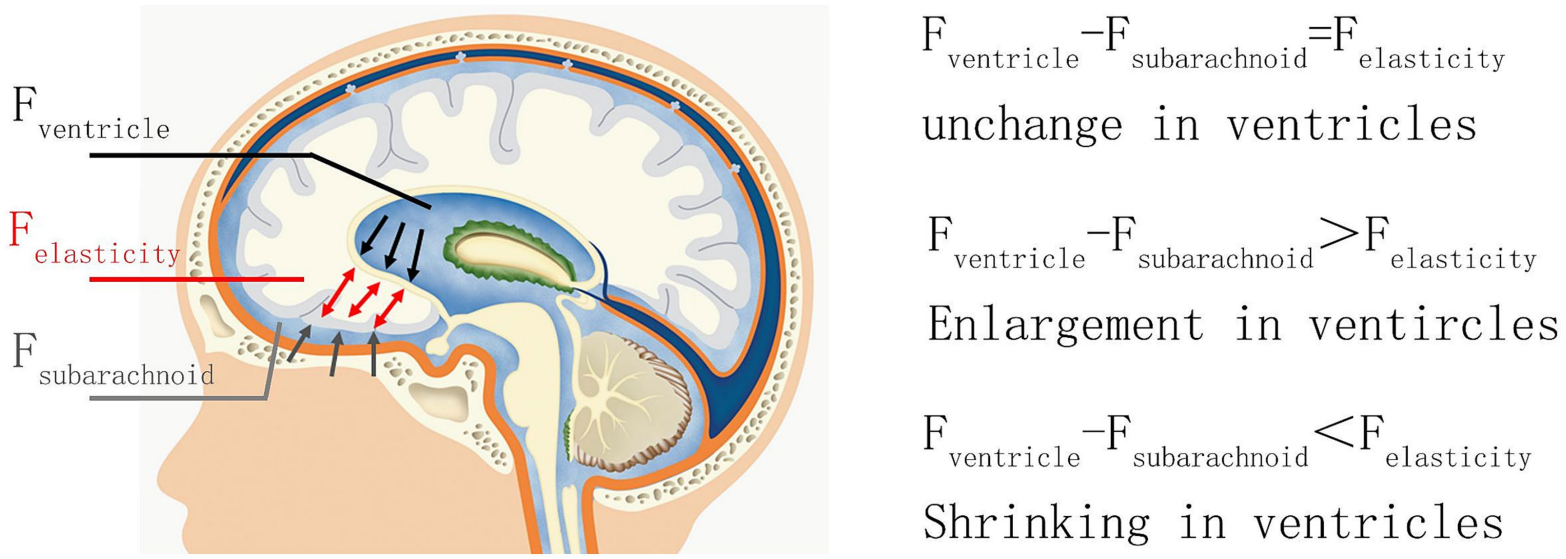
The effect of force from ventricle, subarachnoid space and brain tissue elasticity on ventricular volume.

This study provides a detailed analysis of lateral ventricular subregion volume changes in HANPH patients following VPS, using 3D Slicer-based segmentation. The key findings include: (1) significant reductions in absolute volumes of all lateral ventricular subregions post-VPS, with the frontal horn, temporal horn, and atrium showing strong negative correlations with early clinical improvement; (2) an increase in the relative volume of the lateral body of the lateral ventricle, suggesting higher tissue elasticity in functionally critical regions; and (3) logistic regression indicating that volume reductions in specific subregions (e.g., atrium) significantly increase the likelihood of clinical improvement. These results highlight the importance of localized ventricular changes in predicting treatment response, offering new insights into HANPH pathophysiology (Figure 5).

### 5.1 Theoretical hypotheses for ventricular enlargement in HANPH

The athophysiology of ventricular enlargement in hemorrhagic adult normal pressure hydrocephalus (HANPH) remains incompletely understood (11). While impaired CSF absorption is a commonly proposed mechanism, it fails to explain the persistence or progression of ventriculomegaly under normal intracranial pressure (6). The brain tissue compression and adaptive response hypothesis offers a more comprehensive model (6, 12). It posits that ventricular size is determined by a balance of forces: the intraventricular CSF pressure (F_ventricle), the subarachnoid CSF pressure (F_subarachnoid), and the elastic rebound of brain tissue (F_elasticity). In HANPH, a proposed three-stage process occurs: An initial increase in F_ventricle causes ventricular expansion. This expansion compresses brain tissue, reducing its interstitial fluid and blood flow, which in turn lowers F_elasticity. Subsequently, even if F_ventricle normalizes, the now-reduced F_elasticity prevents ventricular reversion, leading to persistent enlargement (6). Thus, ventricular volume is an indirect marker of these biomechanical changes in brain tissue (12). The translation of these biomechanical shifts into clinical improvement is likely mediated by specific tissue-level mechanisms. Ventricular expansion compresses and stretches periventricular white matter and is associated with reduced regional cerebral blood flow, with the most pronounced perfusion reduction occurring adjacent to the ventricles, thereby compromising metabolically vulnerable long-range white matter pathways (13). In parallel, idiopathic normal-pressure hydrocephalus has been linked to impaired interstitial fluid and glymphatic clearance, as demonstrated by delayed tracer enrichment and reduced clearance on glymphatic MRI, and these fluid-homeostatic disturbances may involve aquaporin-4-dependent water transport at astrocytic endfeet (14). VPS-induced volume reduction may therefore reverse intermediary processes, including periventricular hypoperfusion, interstitial edema, and microstructural white matter disruption; previous perfusion and diffusion studies have shown postoperative or CSF-drainage-related changes that parallel clinical improvement in iNPH. These mechanisms provide biological plausibility for why localized volume reductions—particularly in the frontal horn, temporal horn, and atrium, which are anatomically adjacent to frontal-subcortical, motor, sensory, and associative white matter pathways—correlate preferentially with gait and functional recovery (15). Accordingly, subregional volumetric changes may serve as macroscopic surrogates for microscopic restoration of perfusion, ISF homeostasis, and white matter integrity, supporting their value as indirect biomarkers of treatment response. Clinically, subcompartmental analysis is crucial, as temporal horn enlargement can predict secondary hydrocephalus earlier than frontal horn-based indices like the Evans Index (16), highlighting the significance of regional ventricular changes.

### 5.2 The segmentation and measurement methods of the lateral ventricular subregions

**Figure 6:**
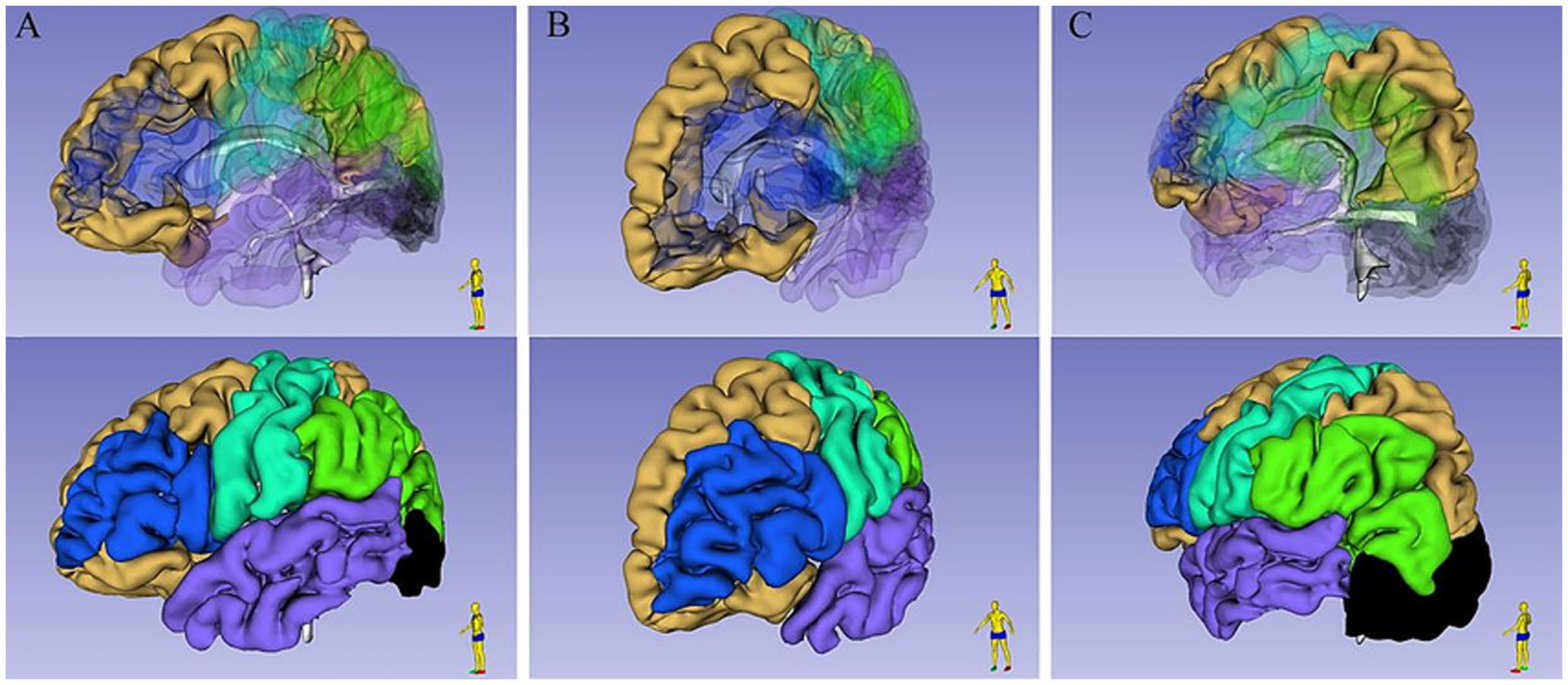
Perspective view of relationship between left lateral ventricle subregions and brain surface tissue. We can see the relationship between left lateral ventricle subregions and brain surface tissue in left lateral view **(A)**, left anterolateral view **(B)** and left posterolateral view **(C)**. Ventricle system was white, frontal lobe corresponds to the frontal horn of the lateral ventricle was blue, Rolandic lobe was light blue, inferior parietal lobe was green, occipital lobe was black, temporal lobe was purple.

The lateral ventricle (LV) can be divided into five distinct subregions: the frontal horn, body, occipital horn, temporal horn, and atrium. The frontal horn is anatomically surrounded by the frontal lobe, with its surface projection corresponding to the triangular and cap areas of the superior and inferior frontal gyri. The body of the LV lies beneath the lower portions of the precentral and postcentral gyri. The atrium forms a confluence where the body, occipital horn, and temporal horn meet, and its surface projection broadly covers the transverse temporal gyrus, angular gyrus, and supramarginal gyrus (Figure 6). While the frontal horn, body, and temporal horn are typically prominent in normal individuals, all five subregions become distinctly visible on imaging in hydrocephalus due to ventricular enlargement, allowing for detailed segmentation with tools like 3D Slicer.

The Frankfurt horizontal (FH) plane, defined by the bilateral external auditory meatus and the infraorbital margin, served as a standard reference plane for this study. It is critical for ensuring consistent anatomical localization in medical imaging and surgical planning (17). During LV segmentation, the frontal horn and body were demarcated by a plane perpendicular to the FH plane that passes through the Monro foramen (Figure 1A). The occipital horn was identified by drawing a perpendicular line from the inferior end of the parieto-occipital sulcus to the FH plane (Figure 1C). The atrium, lacking a distinct anatomical boundary, was defined in 3D reconstructions using the lateral ventricle’s bend point as its apex, with its boundary against the occipital horn marked by a sagittal projection line and segmented in the lateral view (Figure 1G) (18). The remaining portions were automatically assigned to the body and temporal horn. All volumetric measurements were subsequently performed using the “Segment Statistics” module (10).

### 5.3 Relationship between changes in lateral ventricular subregion volumes during the perioperative period of VPS and early clinical improvement in HANPH

The optimal placement of the ventricular catheter during VPS remains inconclusive. Previous studies have supported the use of the frontal horn of the lateral ventricle (19–21), temporal horn (22), atrium (23), and even the occipital horn (24) as potential catheter insertion sites. These findings align with the results of our study, which suggest that the frontal horn, temporal horn, and atrium—especially the RLV subregions—are associated with early clinical improvement in HANPH patients.

The patency of the shunt is crucial to the effectiveness of VPS (25), and the encasement of the choroid plexus is one of the primary causes of shunt obstruction, leading to VPS failure. As a result, most studies currently recommend puncturing the frontal horn of the lateral ventricle toward the interventricular foramen, as this region is free from choroid plexus tissue and is less prone to obstruction, with the added benefit of a larger area and higher tolerance. However, based on the findings of this study, effective drainage of the lateral ventricular atrium while avoiding choroid plexus obstruction and damage to the surrounding critical brain tissue in the atrium may be more beneficial for improving early clinical symptoms in HANPH patients. Based on this hypothesis, the study suggests that puncturing the occipital horn of the lateral ventricle may be superior to puncturing the frontal horn in future research, which was supported by a few existing studies (21). In this study, the volume segmentation and measurement of the lateral ventricular atrium, with its apex at the curved bend of the LV and its base at the boundary with the occipital horn, showed a positive correlation (Figure 1G). Therefore, placing the ventricular catheter at the occipital horn may reduce the likelihood of choroid plexus obstruction, minimize microtrauma to the cortical tissue projected by the atrium during puncture, and more effectively reduce the volume of the atrium compared to catheter placement in the body of the lateral ventricle, achieving a threefold benefit. However, the occipital horn is relatively small and lacks distinct anatomical landmarks, which makes manual puncture less precise, difficultly applied in somewhere without neurological navigation equipment, presenting practical challenges. A study by Zhou et al. (21) also noted that catheter insertion into the occipital horn of the lateral ventricle was performed under navigation guidance, highlighting the need for prospective, randomized comparisons of frontal versus occipital horn catheterization—ideally with neuro-navigation—to determine whether occipital placement truly confers superior clinical outcomes.

The study of lateral ventricular subregions revealed that DaRFV, DaRAV, DaRTV, and DaLAV significantly impact early clinical improvement in HANPH, with the changes in the volume of the lateral ventricular atrium playing a crucial role in symptom improvement. Importantly, these volume changes should be understood as indirect markers of the underlying restorative processes—namely, the re-expansion of compressed periventricular parenchyma, normalization of white matter microarchitecture, and alleviation of mechanical distortion on motor and cognitive pathways—rather than as direct measures of neuronal recovery. The atrium, situated at the confluence of the body, occipital horn, and temporal horn, is anatomically positioned to reflect global alterations in ventricular geometry and periventricular tissue compliance; its volume reduction may thus indirectly signal the relief of biomechanical stress on functionally eloquent regions, including the supramarginal gyrus and angular gyrus. Puncturing the occipital horn to reduce the volume of the atrium may be more beneficial for improving clinical symptoms in HANPH patients, providing new insights for future research directions.

This study has several limitations. First, its retrospective and non-randomized design inherently limits causal inference; although the self-controlled analysis mitigates some confounding, unmeasured confounders and selection bias may still influence the observed associations. A propensity score-adjusted analysis would have been preferable to control for baseline imbalances between groups, and this should be considered in future studies. Second, the grouping was based on clinical decisions rather than randomization, leading to baseline differences between groups. Third, while a post-hoc power analysis confirmed that our final sample size was adequate to detect the main effects, the subgroup analyses, particularly for the smaller groups [e.g., *n* = 33 for HANPH (DC, LSC)], may have limited power to detect more subtle associations. Future prospective studies with larger, multicentric cohorts are warranted to validate these findings and explore subgroup-specific effects with greater precision. Fourth, the two-week follow-up period limits the interpretability of our findings regarding sustained clinical benefit. While early improvement is a recognized predictor of long-term shunt efficacy in hydrocephalus, it does not capture delayed complications (e.g., shunt malfunction, infection, or overdrainage), functional plateau, or late cognitive decline. The absence of mid-term (e.g., 3–6 months) or long-term (≥12 months) outcome data restricts our ability to determine whether the observed volumetric changes predict durable functional recovery or merely transient postoperative effects. Future prospective studies should incorporate serial assessments at 3, 6, and 12 months post-VPS to validate the long-term clinical relevance of these subregional volume changes.

## 6 Conclusion

This pioneering study used 3D Slicer to segment lateral ventricles, revealing that periventricular brain elasticity is linked to functional significance, with key areas like the lateral ventricle body being more elastic. Volume changes showed ipsilateral subregions expand or contract proportionally, while contralateral sides are influenced by shunt placement.

Crucially, clinical improvement was strongly associated with reduced atrial volume, which is influenced by the occipital horn. This suggests a novel hypothesis: direct drainage of the atrium or occipital horn—potentially via navigated occipital catheterization—could optimize outcomes by more effectively targeting this critical region, reducing trauma, and minimizing obstruction risk. This hypothesis requires prospective validation through comparative trials of catheter placement sites (frontal versus occipital horn), ideally with neuro-navigation and standardized volumetric follow-up, before any clinical recommendation can be made.

## Data Availability

The original contributions presented in the study are included in the article/Supplementary material, further inquiries can be directed to the corresponding author.

## Footnotes

1
https://www.slicer.org
