# Supplementary material for: Atrial volume reduction correlates with early improvement in hemorrhage-associated normal pressure hydrocephalus—a 3D computed tomography volumetric study
Source: Front Neurol. 2026 Jul 1;17:1753739. doi: 10.3389/fneur.2026.1753739 (PMC13371204; doi:10.3389/fneur.2026.1753739)
Supplement: Supplementary file 1 [file Supplementary_file_1.DOCX]

**Supplementary methods:**

**Measurement of Cranial and Lateral Ventricular Subregion Volumes Using 3D Slicer**

After completing the previous steps, the “Segment Editor” module was used to create a mask using the “threshold” tool, setting the range to -10 to 10 or 20 to cover the entire ventricle.

Marking the Frontal and Temporal Horns of the Lateral Ventricle (Figure 1A): The three-axis coordinates were adjusted to align the coronal line in the sagittal and axial views and the axial line in the coronal view at the Monro foramen. Segment_1 marked the third ventricle in the three axial views, Segment_2 marked the fourth ventricle in the sagittal view, and Segment_4 and Segment_5 marked the lateral ventricles in the coronal and axial views, respectively, before the Monro foramen, i.e., the frontal horns of the left and right lateral ventricles. Segment_6 and Segment_7 marked the temporal horns of the left and right lateral ventricles in the coronal view, while Segment_3 marked the periventricular tissues.

Marking the Body of the Lateral Ventricle (Figure 1B): The coronal view was moved back one slice. Segment_1 marked the third ventricle in the coronal view, and Segment_6 and Segment_7 marked the body of the left and right lateral ventricles in the coronal view. Segment_3 marked the periventricular tissues.

Marking the Occipital Horn of the Lateral Ventricle (Figure 1C): The coronal line in the sagittal view was positioned at the bottom of the parieto-occipital sulcus. Segment_8 and Segment_9 marked the occipital horns of the left and right lateral ventricles in the coronal view. Segment_3 marked the periventricular tissues.

Marking the Atrium of the Lateral Ventricle (Figure 1D): The coronal view was moved forward one slice. Segment_6 and Segment_7 marked the left and right lateral ventricles in the axial and coronal views, i.e., the atrium of the left and right lateral ventricles. Segment_3 marked the periventricular tissues.

The “Grow from Seeds” tool expands the various subregions of the lateral ventricle (Figure 1E): Each segment is enclosed by Segment_3. Using the “Grow from Seeds” tool, expand nine colors. After previewing (Initialize), click Apply directly and delete the Segment_3 layer. Most stray “burrs” around the edges can be resolved using the “Islands” tool's Keep selected island option. Unresolved areas can be trimmed with the ‘Scissors’ tool, and partial gaps can be repaired using the “Paint” tool with the corresponding layer color.

Expanding the Subregions of the Lateral Ventricle Using the “Grow from Seeds” Tool (Figure 1E): Each segment was closed with Segment_3, and the "Grow from Seeds" tool was used to expand the 9 segments, previewed (initialized), and then applied. The Segment_3 layer was deleted. The scattered “spurs” were addressed using the "Islands", and the remaining issues were trimmed using the "Scissors". Missing areas were repaired using the “Paint” with the corresponding segment color.

Separating the Atrium of the Lateral Ventricle (Figure 1F–G): The "Smoothing" tool with "Closing" (fill holes) was used to fill in any cavities within the segments. As the body, atrium, and temporal horns of the lateral ventricle were still within the same color segments (Segment_6 and Segment_7), the left atrium was separated by identifying the most curved point of the bend between the body and temporal horn. The two intersection points connecting the atrium and occipital horn formed the left atrium. The "Logical Operator" and "Scissors" tools were then used to separate the atrium.

Separating the Body and Temporal Horns of the Lateral Ventricle (Figure 1H–I): New segments (Segment_10 and Segment_11) were added. Following the outlined steps, the "Logical Operator" and "Scissors" tools were used to separate the left lateral temporal horn and body. The same approach was applied to the right lateral ventricle, separating the atrium, temporal horn, and body. The "Wrap Solidify" tool was used to optimize each segment, filling gaps in the layers to prevent measurement errors during volume assessment.

Finally, the “Segment Statistics” module was used to measure the ventricular volume: The “Segment Statistics” module was opened (Step 1), the Scalar Volume option was selected for the data name (Step 2), "Apply" was clicked (Step 3), and the volumes of the various ventricular subregions were obtained (Step 4).

To ensure methodological robustness, we performed a sensitivity analysis using an alternative boundary definition for the atrium. In this alternative approach, the atrium-occipital horn boundary was defined by the midpoint between the splenium of the corpus callosum and the calcarine sulcus on the sagittal plane, rather than the lateral ventricle's bend point. Volumes were remeasured in 30 randomly selected cases (approximately 17% of the total cohort) using this alternative definition. The correlation between primary and alternative atrium volume measurements was assessed using Pearson correlation, and logistic regression was repeated using the alternative atrium volume differences (DaRAV and DaLAV) to confirm the consistency of effect estimates.

**Statistical Analysis**

The absolute ventricular volume is defined as the ratio of the ventricular volume to IV. For example, the absolute volume of the right lateral ventricle (aRLV) is the ratio of the right lateral ventricle volume to IV. The relative ventricular volume is defined as the ratio of the ventricular volume to the whole ventricular volume (WV). For example, the relative volume of the right lateral ventricle (rRLV) is the ratio of the right lateral ventricle volume to WV. The difference in absolute ventricular volume before and after VPS is calculated as the change in absolute volume between the two weeks post-VPS and pre-VPS. For instance, the difference in the absolute volume of the right lateral ventricle (DaRLV) is the difference in the right lateral ventricle volume before and after VPS. Similarly, the difference in relative ventricular volume before and after VPS is calculated as the change in relative volume between the two weeks post-VPS and pre-VPS. For example, the difference in the relative volume of the right lateral ventricle (DrRLV) is the difference in the relative volume of the right lateral ventricle before and after VPS. Other ventricular subregions were followed the same format (abbreviations for other ventricular subregions are provided in "Supplementary Table 1").

A pre-post design was used, which does not require baseline data matching. Categorical data were analyzed using Pearson's chi-square test, with frequencies (n) reported. Continuous data are expressed as mean ± standard deviation (X S). Comparisons of the absolute and relative ventricular volumes before and after VPS were made using the Wilcoxon signed-rank test. The relationship between the differences in absolute and relative ventricular volumes and early clinical improvement was assessed using binary logistic regression analysis. All statistical analyses were performed using SPSS version 25.0 (IBM Corporation), with a p-value ≤ 0.05 considered statistically significant.

The "absolute volume" of a ventricular subregion (e.g., aRFV), defined as the subregion volume divided by the intracranial volume (ICV). The "relative volume" of a subregion (e.g., rRFV), defined as the subregion volume divided by the total lateral ventricular volume (WV), represents the within-ventricle volume partition of that subregion.

**Sample Size Consideration**

Given the retrospective nature of this study, a formal prospective sample size calculation was not performed prior to patient enrollment. The initial sample comprised all eligible HANPH patients who underwent VPS at our institution during the specified study period and met the inclusion and exclusion criteria, resulting in a total of 180 participants.

To assess the statistical power of the study with the achieved sample size, a post-hoc power analysis was conducted using G*Power software (version 3.1.9.7). The analysis was configured for a logistic regression model, based on the key findings from Table S1. The input parameters were as follows: an odds ratio (OR) of approximately 2.8 (a conservative estimate based on the lowest significant OR for DaRFV), a reference event probability (for early improvement) of 0.63 (113/180), an alpha error probability of 0.05, and a total sample size of 180. The analysis considered the four primary predictor variables (DaRFV, DaRAV, DaRTV, DaLAV) as part of the model. This post-hoc analysis indicated that the study achieved a statistical power (1-β) of greater than 90% to detect the observed effects, suggesting that the sample size of 180 was adequate for the primary analyses performed.

**Supplementary Table 1 Abbreviations**

| Abbreviations | Full English name |
| --- | --- |
| **HANPH** | hemorrhage associated normal pressure hydrocephalus |
| **RLV** | right lateral ventricle |
| **LLV** | left lateral ventricle |
| **3^rd^V** | the third ventricle |
| **4^th^V** | the fourth ventircle |
| **VPS** | ventriculoperitoneal shunt |
| **LV** | lateral ventricle |
| **DICOM** | digital Imaging and communications in medicine |
| **DC** | decompressive craniectomy |
| **RSC** | right side catheterization |
| **LSC** | left side catheterization |
| **mRS** | modified Rankin Scale |
| **BI** | Barthel Index |
| **FH** | Frankfurt horizontal plane |
| **SML** | supraorbitomeatal line |
| **OMBL** | orbitomeatal base line |
| **RBL** | Reid’s base line |
| **EI** | Evans' Index |
| **BCI** | Bicaudate Index |
| **IV** | intracranial volume |
| **aRLV** | absolute volume of right lateral ventricle |
| **aLLV** | absolute volume of left lateral ventricle |
| **a3^rd^V** | absolute volume of the third ventricle |
| **a4^th^V** | absolute volume of the fourth ventricle |
| **aLV** | absolute volume of lateral ventricle |
| **aWV** | absolute volume of the whole ventricle |
| **WV** | the whole ventricle |
| **rRLV** | relative volume of right lateral ventricle |
| **rLLV** | relative volume of left lateral ventricle |
| **r3^rd^V** | relativevolume of the third ventricle |
| **r4^th^V** | relative volume of the fourth ventricle |
| **rLV** | relative volume of lateral ventricle |
| **DaRLV** | difference in absolute ventricular volume of right lateral ventricle before and after VPS |
| **DaLLV** | difference in absolute ventricular volume of left lateral ventricle before and after VPS |
| **Da3^rd^V** | difference in absolute ventricular volume of the third ventricle before and after VPS |
| **Da4^th^V** | difference in absolute ventricular volume of the fourth ventircle before and after VPS |
| **DaWV** | difference in absolute ventricular volume of the whole ventricle before and after VPS |
| **DrRLV** | difference in relative ventricular volume of right lateral ventricle before and after VPS |
| **DrLLV** | difference in relative ventricular volume of left lateral ventricle before and after VPS |
| **Dr3^rd^V** | difference in relative ventricular volume of the third ventricle before and after VPS |
| **Dr4^th^V** | difference in relative ventricular volume of the fourth ventircle before and after VPS |
| **1:1 PSM** | the 1:1 propensity score matching |
| **ns** | no significance |
| **CI** | confidence interval |
| **RFV** | frontal horn of right lateral ventricle |
| **RBV** | body of right lateral ventricle |
| **RAV** | atrium of right lateral ventricle |
| **ROV** | occipital horn of right lateral ventricle |
| **RTV** | temporal horn of right lateral ventricle |
| **LFV** | frontal horn of left lateral ventricle |
| **LBV** | body of left lateral ventricle |
| **LAV** | atrium of left lateral ventricle |
| **LOV** | occipital horn of left lateral ventricle |
| **LTV** | temporal horn of left lateral ventricle |
| **aRFV** | absolute volume of frontal horn of right lateral ventricle |
| **aRBV** | absolute volume of body of right lateral ventricle |
| **aRAV** | absolute volume of atrium of right lateral ventricle |
| **aROV** | absolute volume of occipital horn of right lateral ventricle |
| **aRTV** | absolute volume of temporal horn of right lateral ventricle |
| **aLFV** | absolute volume of frontal horn of left lateral ventricle |
| **aLBV** | absolute volume of body of left lateral ventricle |
| **aLAV** | absolute volume of atrium of left lateral ventricle |
| **aLOV** | absolute volume of occipital horn of left lateral ventricle |
| **aLTV** | absolute volume of temporal horn of left lateral ventricle |
| **rRFV** | relative volume of frontal horn of right lateral ventricle |
| **rRBV** | relative volume of body of right lateral ventricle |
| **rRAV** | relative volume of atrium of right lateral ventricle |
| **rROV** | relative volume of occipital horn of right lateral ventricle |
| **rRTV** | relative volume of temporal horn of right lateral ventricle |
| **rLFV** | relative volume of frontal horn of left lateral ventricle |
| **rLBV** | relative volume of body of left lateral ventricle |
| **rLAV** | relative volume of atrium of left lateral ventricle |
| **rLOV** | relative volume of occipital horn of left lateral ventricle |
| **rLTV** | relative volume of temporal horn of left lateral ventricle |
| **DaRFV** | difference in absolute ventricular volume of frontal horn of right lateral ventricle before and after VPS |
| **DaRBV** | difference in absolute ventricular volume of body of right lateral ventricle before and after VPS |
| **DaRAV** | difference in absolute ventricular volume of atrium of right lateral ventricle before and after VPS |
| **DaROV** | difference in absolute ventricular volume of occipital horn of right lateral ventricle before and after VPS |
| **DaRTV** | difference in absolute ventricular volume of temporal horn of right lateral ventricle before and after VPS |
| **DaLFV** | difference in absolute ventricular volume of frontal horn of left lateral ventricle before and after VPS |
| **DaLBV** | difference in absolute ventricular volume of body of left lateral ventricle before and after VPS |
| **DaLAV** | difference in absolute ventricular volume of atrium of left lateral ventricle before and after VPS |
| **DaLOV** | difference in absolute ventricular volume of occipital horn of left lateral ventricle before and after VPS |
| **DaLTV** | difference in absolute ventricular volume of temporal horn of left lateral ventricle before and after VPS |
| **DrRFV** | difference in relative ventricular volume of frontal horn of right lateral ventricle before and after VPS |
| **DrRBV** | difference in relative ventricular volume of body of right lateral ventricle before and after VPS |
| **DrRAV** | difference in relative ventricular volume of atrium of right lateral ventricle before and after VPS |
| **DrROV** | difference in relative ventricular volume of occipital horn of right lateral ventricle before and after VPS |
| **DrRTV** | difference in relative ventricular volume of temporal horn of right lateral ventricle before and after VPS |
| **DrLFV** | difference in relative ventricular volume of frontal horn of left lateral ventricle before and after VPS |
| **DrLBV** | difference in relative ventricular volume of body of left lateral ventricle before and after VPS |
| **DrLAV** | difference in relative ventricular volume of atrium of left lateral ventricle before and after VPS |
| **DrLOV** | difference in relative ventricular volume of occipital horn of left lateral ventricle before and after VPS |
| **DrLTV** | difference in relative ventricular volume of temporal horn of left lateral ventricle before and after VPS |

**Supplementary Table 2 A comparison of the absolute and relative volumes of the lateral ventricle subventricular area (LVSA) in HANPH patients before and after VPS surgery**

| Supplement Table 2 Comparison of the ventricular volume in the lateral ventricle subgroups before and after VPS in HANPH. | | | | | | | | | | | | | | | |
| --- | --- | --- | --- | --- | --- | --- | --- | --- | --- | --- | --- | --- | --- | --- | --- |
| subgroup | n | ventricular volume of the lateral ventricle subgroups | before VPS | | | | | | after VPS 2W | | | | | | Z |
|  |  |  | X±S | 95%CI | | Median | Min | Max | X±S | 95%CI | | Median | Min | Max |  |
| HANPH (non-DC, RSC) | 72 | aRFV(‰) | 14.34±0.65 | 13.04 | 15.64 | 13.75 | 7.43 | 28.96 | 8.12±0.53 | 7.07 | 9.18 | 7.70 | 1.63 | 21.68 | -7.256a*** |
|  |  | aRBV(‰) | 15.09±0.66 | 13.76 | 16.42 | 14.68 | 6.13 | 28.95 | 12.37±0.70 | 11.00 | 13.78 | 11.65 | 1.49 | 24.13 | -3.345a*** |
|  |  | aRAV(‰) | 4.53±0.23 | 4.08 | 4.99 | 4.20 | 1.63 | 9.35 | 2.65±0.19 | 2.27 | 3.02 | 2.38 | 0.00 | 5.17 | -6.914a*** |
|  |  | aROV(‰) | 2.31±0.27 | 1.77 | 2.85 | 1.81 | 0.00 | 8.12 | 0.64±0.08 | 0.47 | 0.81 | 0.42 | 0.00 | 3.01 | -6.066a*** |
|  |  | aRTV(‰) | 8.22±0.44 | 7.33 | 9.10 | 7.30 | 1.57 | 16.03 | 5.15±0.52 | 4.12 | 6.18 | 4.23 | 0.00 | 16.78 | -5.135a*** |
|  |  | aLFV(‰) | 14.85±0.54 | 13.78 | 15.93 | 15.17 | 6.96 | 27.37 | 10.17±0.53 | 9.12 | 11.22 | 10.47 | 1.93 | 19.56 | -5.713a*** |
|  |  | aLBV(‰) | 16.53±0.66 | 15.20 | 17.85 | 16.81 | 7.67 | 26.47 | 14.79±0.69 | 13.41 | 16.18 | 13.95 | 2.47 | 25.04 | -2.161a** |
|  |  | aLAV(‰) | 6.00±0.33 | 5.35 | 6.65 | 5.57 | 1.74 | 14.93 | 3.74±0.25 | 3.23 | 4.24 | 3.64 | 0.29 | 8.62 | -6.263a*** |
|  |  | aLOV(‰) | 4.80±0.47 | 3.87 | 5.73 | 3.72 | 0.11 | 19.18 | 1.91±0.20 | 1.51 | 2.31 | 1.55 | 0.00 | 7.54 | -6.330a*** |
|  |  | aLTV(‰) | 7.83±0.37 | 7.09 | 8.56 | 7.39 | 1.38 | 16.87 | 5.68±0.41 | 4.88 | 6.49 | 5.04 | 0.00 | 12.94 | -3.597a*** |
|  |  | rRFV(%) | 32.74±0.89 | 30.96 | 34.52 | 30.44 | 22.82 | 46.56 | 29.67±0.98 | 27.72 | 31.61 | 29.32 | 16.83 | 52.55 | -3.154a*** |
|  |  | rRBV(%) | 34.09±0.82 | 32.46 | 35.73 | 32.82 | 18.83 | 47.33 | 44.60±0.87 | 42.88 | 46.33 | 44.72 | 25.20 | 58.96 | -6.347b*** |
|  |  | rRAV(%) | 10.21±0.30 | 9.62 | 10.81 | 10.05 | 4.73 | 14.77 | 8.69±0.37 | 7.94 | 9.43 | 8.78 | 0.00 | 14.31 | -3.434a*** |
|  |  | rROV(%) | 4.75±0.47 | 3.81 | 5.70 | 3.72 | 0.00 | 14.64 | 1.88±0.21 | 1.47 | 2.30 | 1.39 | 0.00 | 5.55 | -4.977a*** |
|  |  | rRTV(%) | 18.20±0.63 | 16.94 | 19.46 | 18.72 | 9.00 | 28.05 | 15.16±1.06 | 13.05 | 17.28 | 14.53 | 0.00 | 32.58 | -2.744a*** |
|  |  | rLFV(%) | 30.58±0.77 | 29.04 | 32.11 | 32.17 | 17.48 | 45.35 | 28.95±0.82 | 27.32 | 30.58 | 30.43 | 13.43 | 39.42 | -1.218a |
|  |  | rLBV(%) | 33.41±0.77 | 31.87 | 34.95 | 33.17 | 13.27 | 42.85 | 41.92±0.71 | 40.50 | 43.34 | 40.70 | 32.27 | 54.27 | -6.117b*** |
|  |  | rLAV(%) | 11.80±0.38 | 11.03 | 12.56 | 11.04 | 5.33 | 20.22 | 9.71±0.32 | 9.07 | 10.36 | 9.31 | 5.66 | 16.24 | -5.477a*** |
|  |  | rLOV(%) | 8.84±0.59 | 7.67 | 10.01 | 9.27 | 0.54 | 22.90 | 5.08±0.38 | 4.32 | 5.84 | 4.79 | 0.00 | 12.96 | -5.387a*** |
|  |  | rLTV(%) | 15.38±0.38 | 14.61 | 16.15 | 15.39 | 6.69 | 20.97 | 14.34±0.74 | 12.85 | 15.82 | 14.65 | 0.00 | 26.72 | -0.488a |
| HANPH (non-DC, LSC) | 39 | aRFV(‰) | 13.14±0.56 | 12.00 | 14.28 | 12.80 | 6.70 | 19.20 | 10.13±0.76 | 8.60 | 11.66 | 10.00 | 2.90 | 20.50 | -3.586a*** |
|  |  | aRBV(‰) | 15.26±0.65 | 13.94 | 16.57 | 14.80 | 9.20 | 21.60 | 11.68±0.65 | 10.37 | 13.00 | 11.80 | 6.20 | 18.70 | -4.020a*** |
|  |  | aRAV(‰) | 4.44±0.63 | 3.16 | 5.71 | 3.60 | 1.70 | 17.00 | 2.62±0.34 | 1.94 | 3.30 | 2.00 | 0.70 | 8.70 | -2.598a*** |
|  |  | aROV(‰) | 1.77±0.27 | 1.22 | 2.32 | 1.20 | 0.00 | 5.80 | 0.95±0.17 | 0.60 | 1.29 | 0.40 | 0.00 | 3.20 | -2.971a*** |
|  |  | aRTV(‰) | 6.64±0.38 | 5.87 | 7.41 | 5.80 | 3.30 | 9.90 | 4.43±0.48 | 3.46 | 5.41 | 3.30 | 1.30 | 11.80 | -3.644a*** |
|  |  | aLFV(‰) | 14.56±0.67 | 13.20 | 15.91 | 13.40 | 7.60 | 21.50 | 8.43±0.61 | 7.19 | 9.68 | 8.80 | 1.60 | 16.10 | -5.375a*** |
|  |  | aLBV(‰) | 16.77±0.65 | 15.46 | 18.09 | 16.40 | 10.20 | 23.90 | 11.76±0.71 | 10.33 | 13.19 | 11.20 | 4.80 | 20.70 | -4.837a*** |
|  |  | aLAV(‰) | 3.79±0.25 | 3.29 | 4.29 | 3.50 | 1.80 | 7.40 | 2.00±0.16 | 1.68 | 2.32 | 2.40 | 0.30 | 3.30 | -4.567a*** |
|  |  | aLOV(‰) | 1.89±0.25 | 1.39 | 2.38 | 1.90 | 0.10 | 5.80 | 0.56±0.09 | 0.38 | 0.75 | 0.30 | 0.00 | 1.60 | -4.716a*** |
|  |  | aLTV(‰) | 6.92±0.51 | 5.90 | 7.95 | 6.80 | 3.10 | 12.60 | 2.74±0.27 | 2.19 | 3.29 | 2.60 | 0.40 | 6.30 | -4.859a*** |
|  |  | rRFV(%) | 32.49±1.23 | 30.00 | 34.98 | 30.43 | 20.67 | 48.57 | 34.13±1.44 | 31.23 | 37.04 | 34.66 | 23.02 | 48.37 | -1.214a |
|  |  | rRBV(%) | 37.51±1.19 | 35.10 | 39.92 | 38.34 | 26.72 | 53.88 | 40.87±1.30 | 38.24 | 43.49 | 39.45 | 24.45 | 52.06 | -1.633a |
|  |  | rRAV(%) | 9.89±0.90 | 8.06 | 11.72 | 8.83 | 4.87 | 27.28 | 8.05±0.51 | 7.03 | 9.07 | 7.76 | 4.26 | 15.01 | -1.291b |
|  |  | rROV(%) | 3.8±0.45 | 2.88 | 4.72 | 3.94 | 0.00 | 9.27 | 2.90±0.51 | 1.87 | 3.93 | 2.96 | 0.00 | 12.04 | -2.688b*** |
|  |  | rRTV(%) | 16.31±0.85 | 14.60 | 18.02 | 14.84 | 10.05 | 28.28 | 14.05±0.69 | 12.66 | 15.44 | 13.64 | 8.32 | 20.32 | -2.819b*** |
|  |  | rLFV(%) | 33.34±0.97 | 31.38 | 35.29 | 32.56 | 25.50 | 45.04 | 32.83±1.44 | 29.92 | 35.74 | 33.66 | 17.56 | 51.08 | -0.293a |
|  |  | rLBV(%) | 38.55±0.84 | 36.85 | 40.25 | 37.52 | 31.93 | 48.85 | 47.43±1.22 | 44.96 | 49.90 | 46.87 | 35.59 | 66.74 | -4.815b*** |
|  |  | rLAV(%) | 8.47±0.37 | 7.72 | 9.23 | 7.73 | 6.06 | 14.84 | 7.73±0.50 | 6.73 | 8.74 | 7.20 | 4.15 | 13.47 | -0.879a |
|  |  | rLOV(%) | 3.92±0.51 | 2.89 | 4.95 | 3.93 | 0.19 | 12.52 | 1.87±0.25 | 1.35 | 2.38 | 1.63 | 0.00 | 5.20 | -4.523a*** |
|  |  | rLTV(%) | 15.72±0.93 | 13.84 | 17.61 | 15.66 | 6.46 | 25.30 | 10.14±0.59 | 8.94 | 11.34 | 11.63 | 3.40 | 14.58 | -4.187a*** |
| HANPH (DC, RSC) | 36 | aRFV(‰) | 12.97±0.54 | 11.88 | 14.06 | 13.35 | 7.00 | 17.20 | 6.85±0.49 | 5.87 | 7.84 | 7.50 | 0.20 | 11.40 | -4.927a*** |
|  |  | aRBV(‰) | 11.49±0.76 | 9.95 | 13.04 | 10.45 | 5.10 | 20.80 | 9.27±0.80 | 7.66 | 10.89 | 9.00 | 1.00 | 17.20 | -2.406a** |
|  |  | aRAV(‰) | 2.94±0.29 | 2.36 | 3.53 | 2.35 | 0.90 | 6.70 | 1.61±0.19 | 1.22 | 1.99 | 1.40 | 0.00 | 3.90 | -4.441a*** |
|  |  | aROV(‰) | 1.28±0.17 | 0.94 | 1.62 | 1.40 | 0.00 | 3.20 | 0.93±0.18 | 0.56 | 1.29 | 0.40 | 0.00 | 3.00 | -2.197a** |
|  |  | aRTV(‰) | 6.25±0.63 | 4.97 | 7.52 | 5.75 | 0.40 | 15.70 | 3.18±0.31 | 2.54 | 3.82 | 3.4 | 0.00 | 6.20 | -4.009a*** |
|  |  | aLFV(‰) | 17.26±0.78 | 15.67 | 18.86 | 18.05 | 10.90 | 24.40 | 8.14±0.75 | 6.62 | 9.67 | 7.25 | 0.20 | 15.90 | -5.139a*** |
|  |  | aLBV(‰) | 15.94±1.31 | 13.27 | 18.60 | 13.90 | 5.50 | 30.30 | 11.38±0.90 | 9.55 | 13.21 | 10.85 | 1.10 | 19.60 | -2.357a** |
|  |  | aLAV(‰) | 4.61±0.42 | 3.77 | 5.46 | 3.75 | 1.00 | 9.10 | 2.21±0.20 | 1.82 | 2.61 | 2.85 | 0.00 | 3.50 | -4.128a*** |
|  |  | aLOV(‰) | 2.55±0.34 | 1.86 | 3.23 | 2.20 | 0.30 | 7.40 | 1.27±0.16 | 0.94 | 1.60 | 0.95 | 0.00 | 2.70 | -3.232a*** |
|  |  | aLTV(‰) | 7.67±0.93 | 5.78 | 9.56 | 5.00 | 0.30 | 20.70 | 4.59±0.61 | 3.34 | 5.83 | 3.35 | 0.00 | 11.50 | -2.689a*** |
|  |  | rRFV(%) | 39.07±1.48 | 36.07 | 42.07 | 38.80 | 27.59 | 52.32 | 31.87±1.54 | 28.75 | 34.99 | 29.80 | 19.54 | 49.68 | -4.195a*** |
|  |  | rRBV(%) | 33.08±1.09 | 30.87 | 35.29 | 31.51 | 21.46 | 46.63 | 45.07±2.13 | 40.75 | 49.39 | 41.71 | 31.82 | 80.46 | -4.336b*** |
|  |  | rRAV(%) | 7.97±0.36 | 7.24 | 8.70 | 7.76 | 4.74 | 11.40 | 6.30±0.50 | 5.29 | 7.31 | 6.57 | 0.00 | 11.45 | -3.241a*** |
|  |  | rROV(%) | 3.32±0.41 | 2.49 | 4.15 | 3.49 | 0.01 | 7.34 | 3.46±0.60 | 2.24 | 4.68 | 2.29 | 0.00 | 9.69 | -0.094a |
|  |  | rRTV(%) | 16.56±1.12 | 14.29 | 18.83 | 16.10 | 2.94 | 25.96 | 13.30±1.15 | 10.95 | 15.64 | 13.35 | 0.00 | 23.18 | -2.027a** |
|  |  | rLFV(%) | 39.22±1.91 | 35.35 | 43.09 | 39.01 | 19.69 | 60.26 | 28.51±1.62 | 25.22 | 31.79 | 30.14 | 14.05 | 48.78 | -4.430a*** |
|  |  | rLBV(%) | 32.57±1.16 | 30.22 | 34.91 | 31.21 | 22.73 | 48.83 | 43.62±1.89 | 39.79 | 47.46 | 42.60 | 27.95 | 73.50 | -4.242b*** |
|  |  | rLAV(%) | 9.06±0.37 | 8.31 | 9.80 | 8.97 | 5.57 | 12.97 | 7.33±0.57 | 6.17 | 8.49 | 7.58 | 0.00 | 12.68 | -1.414a |
|  |  | rLOV(%) | 4.94±0.57 | 3.79 | 6.10 | 3.88 | 1.14 | 11.32 | 5.62±0.71 | 4.19 | 7.06 | 4.90 | 0.00 | 13.31 | -0.754b |
|  |  | rLTV(%) | 14.21±1.13 | 11.93 | 16.50 | 13.94 | 1.61 | 28.32 | 14.91±1.81 | 11.23 | 18.59 | 13.15 | 0.00 | 39.92 | -0.424b |
| HANPH (DC, LSC) | 33 | aRFV(‰) | 16.01±1.38 | 13.21 | 18.82 | 13.80 | 2.30 | 27.50 | 9.88±0.97 | 7.91 | 11.86 | 8.50 | 1.80 | 20.70 | -4.737a*** |
|  |  | aRBV(‰) | 22.11±1.20 | 19.67 | 24.54 | 25.00 | 9.80 | 33.30 | 15.55±1.20 | 13.11 | 17.98 | 12.50 | 8.30 | 27.50 | -4.102a*** |
|  |  | aRAV(‰) | 7.83±0.84 | 6.12 | 9.54 | 6.70 | 2.30 | 16.80 | 4.75±0.92 | 2.86 | 6.63 | 4.00 | 0.60 | 19.90 | -3.942a*** |
|  |  | aROV(‰) | 3.61±0.49 | 2.60 | 4.62 | 4.40 | 0.30 | 7.60 | 1.49±0.38 | 0.71 | 2.27 | 0.80 | 0.00 | 7.90 | -4.373a*** |
|  |  | aRTV(‰) | 12.13±1.01 | 10.06 | 14.19 | 11.70 | 1.90 | 21.80 | 7.82±0.96 | 5.86 | 9.78 | 8.00 | 2.00 | 18.20 | -4.506a*** |
|  |  | aLFV(‰) | 13.89±1.08 | 11.70 | 16.08 | 12.90 | 4.40 | 26.10 | 8.73±0.85 | 6.99 | 10.46 | 8.40 | 2.70 | 19.80 | -4.979a*** |
|  |  | aLBV(‰) | 15.49±0.94 | 13.58 | 17.40 | 15.50 | 7.50 | 23.20 | 11.17±0.99 | 9.15 | 13.18 | 10.20 | 4.80 | 20.00 | -3.977a*** |
|  |  | aLAV(‰) | 3.77±0.50 | 2.74 | 4.79 | 3.20 | 0.40 | 10.20 | 2.16±0.31 | 1.53 | 2.80 | 1.20 | 0.20 | 4.90 | -3.759a*** |
|  |  | aLOV(‰) | 1.87±0.29 | 1.28 | 2.47 | 1.20 | 0.10 | 5.10 | 0.77±0.16 | 0.45 | 1.10 | 0.10 | 0.00 | 2.60 | -5.022a*** |
|  |  | aLTV(‰) | 7.07±0.84 | 5.36 | 8.77 | 7.00 | 0.50 | 14.70 | 4.02±0.63 | 2.73 | 5.30 | 2.90 | 0.10 | 12.50 | -3.567a*** |
|  |  | rRFV(%) | 26.25±1.65 | 22.88 | 29.62 | 27.75 | 5.06 | 40.78 | 25.98±1.43 | 23.07 | 28.89 | 27.40 | 9.40 | 36.44 | -0.715a |
|  |  | rRBV(%) | 37.72±1.63 | 34.40 | 41.03 | 36.80 | 25.58 | 56.65 | 42.83±1.45 | 39.88 | 45.78 | 42.34 | 27.45 | 53.11 | -3.458b*** |
|  |  | rRAV(%) | 11.91±0.62 | 10.65 | 13.17 | 10.99 | 6.71 | 18.24 | 9.65±0.91 | 7.80 | 11.51 | 9.48 | 3.37 | 21.60 | -2.172a** |
|  |  | rROV(%) | 4.92±0.55 | 3.81 | 6.03 | 6.50 | 1.02 | 9.31 | 2.81±0.44 | 1.92 | 3.70 | 1.65 | 0.02 | 8.63 | -3.513a*** |
|  |  | rRTV(%) | 19.20±1.01 | 17.14 | 21.27 | 18.21 | 7.26 | 27.48 | 18.73±0.74 | 17.22 | 20.24 | 19.28 | 12.40 | 26.91 | -0.509a |
|  |  | rLFV(%) | 34.02±1.27 | 31.43 | 36.61 | 31.55 | 23.32 | 46.42 | 34.29±1.57 | 31.09 | 37.50 | 33.20 | 24.10 | 56.34 | -0.777a |
|  |  | rLBV(%) | 39.26±1.10 | 37.03 | 41.49 | 39.24 | 29.00 | 48.80 | 44.45±1.42 | 41.57 | 47.33 | 43.87 | 32.19 | 57.23 | -3.976b*** |
|  |  | rLAV(%) | 7.99±0.61 | 6.74 | 9.24 | 8.37 | 2.00 | 14.14 | 6.81±0.56 | 5.67 | 7.95 | 8.27 | 2.45 | 10.99 | -2.922a*** |
|  |  | rLOV(%) | 3.75±0.43 | 2.87 | 4.63 | 3.86 | 0.30 | 7.03 | 2.02±0.33 | 1.35 | 2.70 | 1.04 | 0.00 | 5.95 | -5.017a*** |
|  |  | rLTV(%) | 14.98±1.01 | 12.92 | 17.04 | 15.99 | 2.71 | 21.27 | 12.43±0.92 | 10.55 | 14.31 | 12.26 | 1.70 | 20.86 | -3.082a*** |
| a, Wilcoxon rank test, based on positive rank. b, Wilcoxon rank test, based on negative rank, *P＜0.05, **P＜0.01 and *** P＜0.001 | | | | | | | | | | | | | | | |
